# A Retrospective Case Series of a Novel Spinal Cord Stimulator Trial Technique with Less Displacement and Migration of the Trial Leads

**DOI:** 10.1155/2019/1236430

**Published:** 2019-06-09

**Authors:** N. Shaparin, K. Gritsenko, P. Agrawal, S. Kim, S. Wahezi, A. Gitkind, J. Hascalovici, A. Vydyanathan, J. Bernstein, A. Dizdarevic, N. Mehta, A. Kaufman

**Affiliations:** ^1^Montefiore Multidisciplinary Pain Program, Montefiore Medical Center, Bronx, NY, USA; ^2^Department of Anesthesiology, Montefiore Medical Center, Bronx, NY, USA; ^3^Department of Pain Medicine, Weill Cornell Medicine Center, New York, NY, USA; ^4^Department of Anesthesiology, Rutgers-New Jersey Medical School, Newark, NJ, USA

## Abstract

**Background:**

Spinal cord stimulation is an established treatment option for certain chronic pain conditions which have been previously unresponsive to conservative therapies or potentially for a subset of patients who have not improved following spine surgery. Prior to permanent lead implantation, stimulator lead trials are performed to ensure adequate patient benefit. During these trials, one of the most common complications and reasons for failure is the displacement and migration of the trial leads, resulting in lost therapeutic coverage. Other complications include infection and dislodged bulky dressings. There is a paucity of literature describing an adequate procedural method to prevent these common complications.

**Objective:**

This study utilizes a series of 19 patients to evaluate a new technique for securing percutaneous spinal cord simulator trial leads, which may minimize dislodgement and migration complications and improve the rate of trial success.

**Study Design:**

Retrospective case series.

**Setting:**

New Jersey Medical School, Department of Anesthesiology, Pain Management Division.

**Methods:**

A retrospective chart review was conducted on 19 consecutive patients undergoing placement of the percutaneous thoracic spinal cord stimulator trial leads for pain associated with lumbar spine pathology over a two-year period (2010–2012).

**Results:**

Of the 19 patients in our cohort, there was one trial lead displacement, no lead migrations, and no site infections. Thirteen patients went on to permanent lead implantation. This improved trial lead placement technique had a high success rate with a low number of complications.

**Limitations:**

Small sample size, retrospective case series, and no control group for comparison.

**Conclusion:**

This case series was able to demonstrate that our described novel spinal cord stimulator trial lead placement and dressing technique can decrease the incidence of lead displacement and migration, thus improving trial success.

## 1. Introduction

Spinal cord stimulation (SCS) has been successfully used for more than 3 decades to treat certain chronic pain conditions [1]. It operates on the gate theory principle of pain by stimulating the dorsal column and modulating transmission through the central nervous system [2]. SCS is an effective pain management modality in properly selected chronic pain patients that do not have adequate levels of analgesia with conservative medical therapies and/or where side effects hinder the ability to increase medication doses for the sufficient effect [3]. Its successful use has been described in a variety of neuropathic pain conditions, including failed back surgery syndrome, complex regional pain syndrome, peripheral diabetic nephropathy, and postherpetic neuralgia [4–8]. Conventional SCS requires the insertion of electrodes into the epidural space of the thoracic or cervical spine and adjusting the lead position to the appropriate spinal level in order to provide stimulation that evokes therapeutic paresthesia at the targeted dermatomal level [7]. However, prior to permanent placement of the spinal cord stimulator, a trial is generally performed to ensure that the patient will experience adequate levels of pain relief with this modality.

One of the most common complications associated with percutaneous stimulator trials is lead migration. In fact, the average lead migration for all subjects in one study by Kim et al. from a standing to sitting position was 3.05 mm inferiorly [9]. Other complications include infection, cerebrospinal fluid leak, loss of therapeutic effect, unpleasant paresthesia, and loss of paresthesia [10–12]. Early studies reported percutaneous lead migration rates as high as 69.2% [13]. A 2004 meta-analysis of 2,700 implants by Cameron, which covered 20 years of literature, demonstrated lead migration as being the most common complication with an incidence of 13.2%, lead breakage with an incidence of 9.1%, infection with an incidence of 3.4%, and unwanted stimulation, which may be suggestive of subtle lead displacement, with an incidence of 2.4% [1]. A 2013 review by Bendersky and Yampolsky found lead migration to have an incidence from 11.3% to 13.2% and an infection rate from 2.5% to 14%, with a mean of approximately 5% [14]. Another study by Villavicencio found that 16 of the 27 patients (59%) with permanent electrodes required a total of 36 lead repositioning procedures [15].

Unpublished quality assurance data at the New Jersey Medical School Anesthesiology Pain Center (NJMS APC) found that the most common reason for SCS trial failure was lead displacement and migration due to the dressing becoming dislodged and thus pulling the lead out of the targeted trial position. There have been techniques described in the literature that aim to decrease the rate of lead migration in SCS trials [16]. Unfortunately, evaluation of their efficacy is limited, and none of these techniques have been widely adopted. In this article, we will present a novel SCS trial lead securing technique and outcome data from 19 consecutive patients undergoing spinal cord percutaneous stimulator trials over a two-year period from 2010 through 2012. This technique differs from conventional techniques by relying on subcutaneous tunneling from the entry site, with threading of the lead contralaterally, as well as anchoring sutures at an exit site that is not the original incision site to maintain the lead position. This technique has afforded greater patient comfort, smaller and less bulky dressings, and decreased risk of lead displacement and migration, resulting in more reliable trials.

## 2. Methods

### 2.1. Retrospective Case Series

This is a retrospective case series of chronic pain patients attending a pain clinic in New Jersey. After approval from the institutional review board (IRB) (IRB Approval Number: 2013003120), billing records from 2010 to 2012 were queried using CPT code 63650, “Percutaneous implantation of neurostimulator electrode array, epidural.” All 19 patients identified were included in this case series. Charts were reviewed, and each patient's age, gender, diagnosis, complications of SCS trial, length of trial, and average pretrial pain scale were recorded, and a quantitative analysis was performed.

### 2.2. Novel Spinal Cord Stimulator Trial Lead Placement Technique

**Figure 1:**
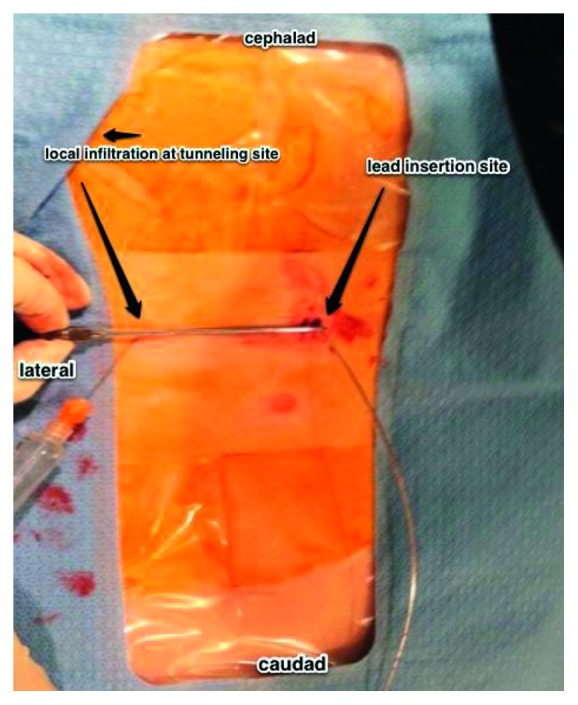
View of the patient's thoracic and lumbar spine region. The lead trial is inserted into the lumbar spine (right side of the image). A mark is then made 6 cm contralateral to the original incision site, and lidocaine 1% is infiltrated (left side of the image).

**Figure 2:**
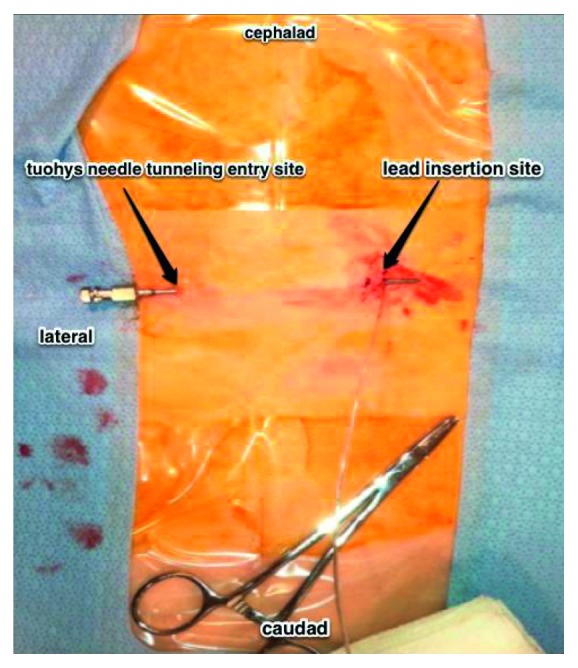
View of the patient's thoracic and lumbar back. The 14-gauge Tuohy needle is progressed subcutaneously until the tip advances through the incision site inferior to the stimulator lead.

**Figure 3:**
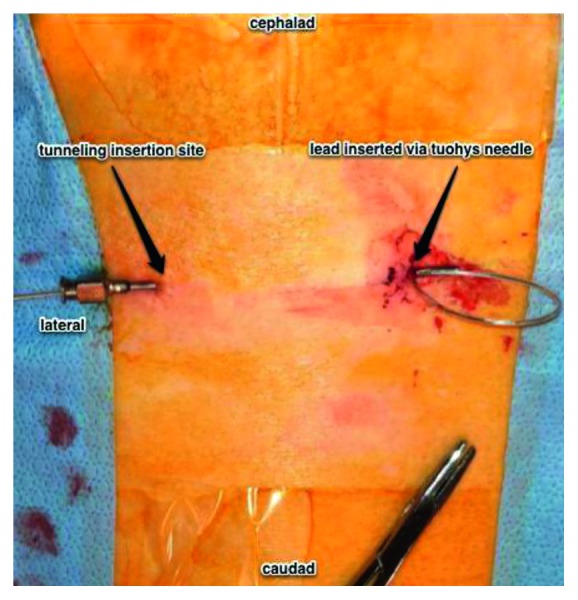
View of the patient's thoracic and lumbar back. The lead is inserted into the Tuohy needle, tunneling through, and exited via the Tuohy needle on the left of image.

**Figure 4:**
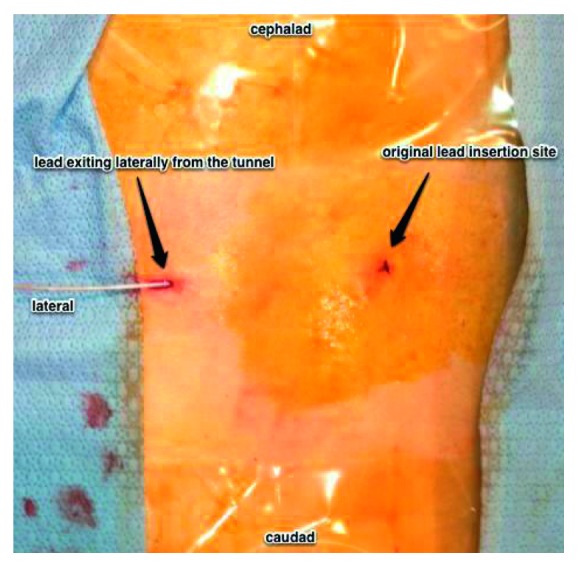
View of the patient's thoracic and lumbar back. The stylet is removed, and the stimulator lead is then guided into the lumen of the needle and is threaded until it exits the proximal end.

**Figure 5:**
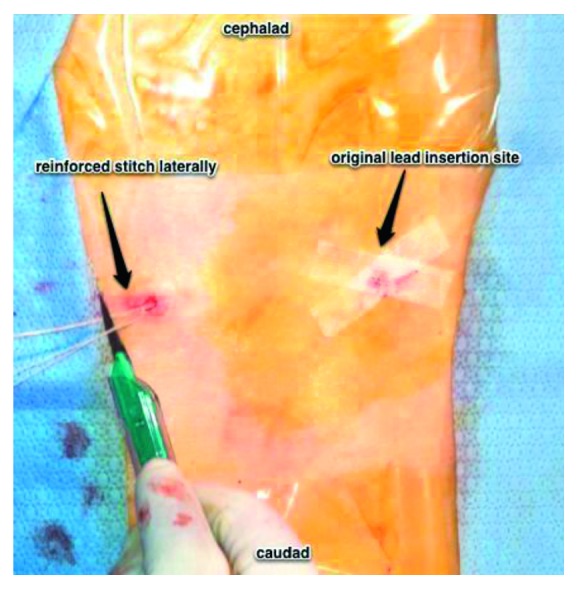
View of the patient's thoracic and lumbar back. The needle is removed, and then while placing gentle traction, the lead is pulled through until the exposed loop is subcutaneous. At this juncture, under fluoroscopy, the lead is carefully pulled caudad to replicate the accepted mapped position. The original incision is then closed using benzoin, steristrips, and covered with sterile gauze and tegaderm. At the new lead exit site, an anchoring suture is placed using a 3-0 nylon suture.

**Figure 6:**
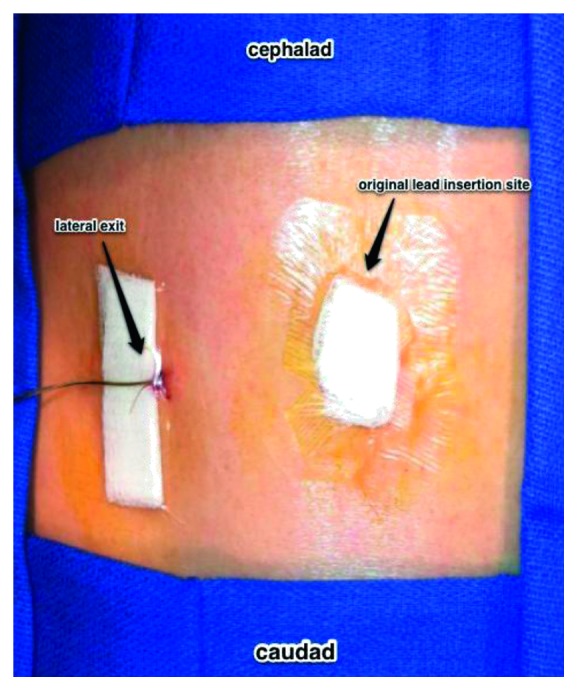
View of the patient's thoracic and lumbar back. A 2″ × 2″ gauze is then folded in half and placed lateral to the lead exit. Benzoin is applied, and the area is covered with tegaderm.

A single physician trialed all patients in the case series. Once the access point had been marked and infiltrated with lidocaine 1%, an incision was made with a #12 scalpel. The 14-gauge modified Tuohy needle was placed through the incision site and then advanced into the epidural space using the loss of the resistance technique, under direct fluoroscopic guidance. The stimulator trial lead was then advanced in the usual manner, positioned adequately to the desired spinal level, and tested to achieve paresthesias in the appropriate distribution. No complications were noted during the placement. For most patients, this was achieved with the tip of the lead at the apex of the T9 vertebral body. Prior to removing the introducer needle, the lead was advanced slightly more cephalad from its mapped position. The introducer needle was removed leaving the lead in place. A mark was then made on the same cephalad-caudad plane 6 cm contralaterally to the original incision site, and lidocaine 1% was infiltrated (Figure 1). The 14-gauge Tuohy needle was then introduced through the skin from the marked site and advanced subcutaneously until the tip appeared through the incision site inferior to the stimulator lead (Figures 2 and 3). The stylet was removed, and the stimulator lead was then guided into the lumen of the epidural needle and was threaded until it exited the proximal end (Figure 4). The needle was removed, and then with placing gentle traction, the exposed loop was pulled into the subcutaneous “tunnel”. At this juncture, under fluoroscopic guidance, the lead was carefully pulled slightly caudad with periodic stimulation testing until the position was obtained to replicate the accepted previously mapped position. The original incision was then closed using benzoin, ½″ × 4″ steristrips, and covered with sterile 2″ × 2″ gauze with a 6 cm × 7 cm tegaderm (Figure 5). At the new lead exit site (bottom of photo), an anchoring suture was placed using a 3-0 nylon suture (Figure 5) and this position at the skin was marked on the lead by a permanent skin marker. A 2″ × 2″ gauze was then folded in half and placed lateral to the lead exit so as to protect the skin from direct pressure. Benzoin was applied, and the area was covered with 6 cm × 7 cm tegaderm (Figure 6).

## 3. Results

The charts of all 19 patients in our study who underwent lumbar spinal cord stimulator trials using the described subcutaneous tunneling technique were reviewed. The demographics demonstrate a mean age of 58.4 years (range 34 to 83 years), with 8 men and 11 women included. The primary diagnoses and indication for the procedure included thirteen patients with postlaminectomy syndrome, two patients with complex regional pain syndrome, one patient each with diabetic neuropathy, arachnoiditis, peroneal nerve neuropathy, and degenerative disk disease. The mean pretrial pain scale was 8.6 (range 6 to 10), and the mean length of the stimulator trial was 3.75 days (range 3 to 8 days). Of the 19 trials, one lead was accidentally removed during the trial period by a nursing assistant. Of the remaining eighteen trials, no lead displacement or migration was noted. Lack of lead displacement was confirmed by noting the previously marked position on the lead to be identical to its originally marked site at the skin. All patients successfully completed the trial. No other complications were noted, and there were no infections.

## 4. Discussion

To our knowledge, this is the first reported case series describing a novel subcutaneous tunneling technique and subsequent anchoring sutures at a contralateral distance from the entry site for percutaneous spinal cord stimulation trials. Despite efforts to minimize its rates, lead migration remains a leading cause of spinal cord stimulation trial failure [1, 14, 17]. These failures lead to inadequate or delayed pain relief for the patient, decreased patient satisfaction, potential repeat SCS trials, and additional costs to the health care system. Bulky dressings are often placed to ensure the stabilization of the trial leads. However, these bulky dressings increase the risk of subsequent lead movement with normal daily activity, movement, and sleep, which inevitably places an increased risk of the leads becoming dislodged. Additionally, as the trial leads are traditionally inserted via a direct line of entry with minimal fixation, they are more prone to migration.

Several studies have tried alternative techniques aimed at diminishing SCS lead migration rates. Osborne et al. found that anchoring trial leads to the skin with suture and tape significantly increased inferior migration compared with anchoring with tape alone [18]. Mironer et al. describe a “midline anchoring” technique where the lead placement stylet was inserted contralaterally, with subsequent crossing of the midline and rotation of the tip to place the electrode. Compared to conventional stylet entry on the ipsilateral side, lead migration in both SCS trial and permanent lead implantation was shown to be significantly reduced [16, 19]. In addition to securing trial leads, studies have tried to secure permanent SCS leads as well. Renard and North reported a single lateral lead migration and no longitudinal lead migration in 99 patients when using silicone elastomer adhesive in permanent SCS lead placement [20]. Kumar et al. suggest the use of silicon glue, the implantation of the generator in the abdominal wall, and the use of a strain relief loop between the anchor and internal pulse generator in order to reduce the tensile load on the lead during changes in body position, as electrode migration occurs when the load on the lead exceeds the capacity of the anchor being used to fix it [21, 22]. Connor et al. found no lead migration in 42 patients when using bone cement at the laminectomy site of SCS lead placement [23] although the use of bone cement with percutaneous lead placement does not appear to be practical. The evaluation of these various approaches for feasibility and efficacy in percutaneous trial lead placements and implantation is limited. This case series provides support for a novel subcutaneous tunneling technique for SCS trial lead placement.

Our described subcutaneous tunneling and anchoring of the lead contralateral to the entry site minimizes the risk of complications. 94.7% of our trials did not have any associated lead migration or complication, apart from the one patient who had an inadvertent lead removal by the patient's nurse aide. This appears to be a marked improvement over the reported rates of lead migration and complications in the literature although our study has a small sample size. Our technique allows for less bulky dressings, which we think decreases the chance for accidental movement of the dressings and subsequent lead displacement. The subcutaneous tunneling also allows for stronger fixation of the leads. The portion of the lead that is tunneled contralateral beneath the skin, as opposed to the traditional direct line of entry, provides necessary slack and tensile strength to withstand tension that is naturally placed on the leads.

Our technique also provides additional benefit to the patients due to the decreased risk of lead migration, the need for a repeat SCS trial, and loss of pain relief. Additionally, there may be potentially decreased risk of infection as well given that a large portion of the lead remains subcutaneous during the trial period. Patient comfort is also enhanced with our dressings that are more comfortable and less bulky. Overall, this leads to an increased chance of the successful trial due to a low rate of complication. Additionally, the placement of the exit site contralateral to the insertion site provides patients with greater comfort and flexibility in regard to connecting the leads to the external pulse generator, as they can situate the generator near the midaxillary line when walking around or lying down.

One of the major limitations of our study is the lack of imaging to confirm the lead position immediately posttrial and prelead removal. However, all of our patients reported that they maintained therapeutic paresthesias during the trial period which implicates a stable lead position. Additionally, nondisplacement was confirmed by noting the marked position on the lead during removal to be identical to the original mark placed at the time of trial. Future studies should also compare different types of leads as well as leads from different manufacturers specifically focusing on number of active contacts in the lead, the edge to edge spacing between the active contacts as well as the contact span distance to see if these variables can affect migration. Lastly, upcoming studies need to take into consideration patient variability in residual excess skin and subcutaneous tissue as is often present in obese and morbidly obese patients evaluating migration rates in patients with different body mass indexes.

While we believe that this technique provides promising results, follow-up prospective studies utilizing larger sample sizes as well as radiographic confirmation of the trial lead position prior to pulling the lead at the end of the trial period are warranted to further establish the efficacy of this tunneling method leading to reduced rates of lead migration.

## 5. Conclusion

Contralateral subcutaneous tunneling and anchoring of SCS trial leads is a promising technique for patients undergoing SCS trials. This study, albeit small in number of patients, demonstrates potentially decreased rates of lead migration and complications as well as high levels of patient comfort and satisfaction. It is a relatively quick and low-risk technique that may decrease lead migration rates, decrease infection risk, increase SCS trial success, and improve patient's pain and comfort. While our center continues to utilize this technique and accrue data for future evaluation, further investigation is warranted. Larger prospective studies are necessary to confirm that our subcutaneous tunneling technique is an improvement over the traditional and alternative techniques currently in practice. Studies evaluating our technique in permanent SCS lead placement may also be beneficial.

## Data Availability

The data used to support the findings of this study are available from the corresponding author upon request.
